# Characterization and Reconstitution of Human Lipoyl Synthase (LIAS) Supports ISCA2 and ISCU as Primary Cluster Donors and an Ordered Mechanism of Cluster Assembly

**DOI:** 10.3390/ijms22041598

**Published:** 2021-02-05

**Authors:** Amber L. Hendricks, Christine Wachnowsky, Brian Fries, Insiya Fidai, James A. Cowan

**Affiliations:** 1 Department of Chemistry and Biochemistry, The Ohio State University, 100 West 18th Ave, Columbus, OH 43210, USA; hendricks.223@osu.edu (A.L.H.); wachnowsky.1@osu.edu (C.W.); fries.94@osu.edu (B.F.); fidai.1@osu.edu (I.F.); 2 The Ohio State Biochemistry Program, The Ohio State University, 100 West 18th Ave, Columbus, OH 43210, USA; 3 The Biophysics Graduate Program, The Ohio State University, 100 West 18th Ave, Columbus, OH 43210, USA

**Keywords:** Fe–S cluster trafficking, lipoyl synthase, [4Fe–4S] cluster proteins, cluster reconstitution

## Abstract

Lipoyl synthase (LIAS) is an iron–sulfur cluster protein and a member of the radical S-adenosylmethionine (SAM) superfamily that catalyzes the final step of lipoic acid biosynthesis. The enzyme contains two [4Fe–4S] centers (reducing and auxiliary clusters) that promote radical formation and sulfur transfer, respectively. Most information concerning LIAS and its mechanism has been determined from prokaryotic enzymes. Herein, we detail the expression, isolation, and characterization of human LIAS, its reactivity, and evaluation of natural iron–sulfur (Fe–S) cluster reconstitution mechanisms. Cluster donation by a number of possible cluster donor proteins and heterodimeric complexes has been evaluated. [2Fe–2S]-cluster-bound forms of human ISCU and ISCA2 were found capable of reconstituting human LIAS, such that complete product turnover was enabled for LIAS, as monitored via a liquid chromatography–mass spectrometry (LC–MS) assay. Electron paramagnetic resonance (EPR) studies of native LIAS and substituted derivatives that lacked the ability to bind one or the other of LIAS’s two [4Fe–4S] clusters revealed a likely order of cluster addition, with the auxiliary cluster preceding the reducing [4Fe–4S] center. These results detail the trafficking of Fe–S clusters in human cells and highlight differences with respect to bacterial LIAS analogs. Likely in vivo Fe–S cluster donors to LIAS are identified, with possible connections to human disease states, and a mechanistic ordering of [4Fe–4S] cluster reconstitution is evident.

## 1. Introduction

Lipoic acid is a vital metabolite that is involved in a diverse set of biochemical roles, such as antioxidants and performing a variety of cellular functions in multiple metabolic pathways. These include decarboxylation reactions involving pyruvate, α-ketoglutarate, the glycine cleavage system, and others [1,2,3,4]. Despite its importance to human health, there has been little biochemical investigation of the eukaryotic enzyme that facilitates the final step of lipoic acid synthesis (lipoyl synthase, LIAS), with the exception of the expression of the porcine homolog and NMR investigation of the human protein and its structural relationship to S-adenosylmethionine (SAM) [5,6]. The biosynthetic step which is facilitated by LIAS is a double sulfur insertion to the sixth and eighth carbons of the octanoyl precursor of lipoic acid. The activity of isolated bacterial LIAS had historically been limited to one turnover in vitro, but multiple turnovers have been observed for the *Escherichia coli* (*E. coli*) enzyme following introduction of iron–sulfur (Fe–S) cluster donor proteins that facilitate reactivation of the auxiliary cluster [7,8]. Presumably, this reflects the need to refresh the auxiliary cluster, which is the source of sulfur for the insertion reactions. The other (reducing) cluster promotes radical formation on S-adenosylmethionine (SAM) and produces a 5’-deoxyadenosine (5’-dA) radical that abstracts hydrogen from the octanoyl sidechain at the site for subsequent sulfur insertion [9].

Members of the radical SAM superfamily share a C–X_3_–C–X_2_–C iron–sulfur cluster binding motif for the reducing [4Fe–4S] cluster that acts as an electron source to produce the radical form of 5’-dA [10,11]. SAM provides the fourth Fe ligand for the reductive cluster [12]. Two equivalents of SAM are required for the double sulfur insertion mediated by LIAS because each produces one radical carbon in the octanoyl substrate; consecutively at the sixth and eighth carbons [13]. The auxiliary [4Fe–4S] cluster binds to a C–X_4_–C–X_5_–C motif exclusive to lipoyl synthases and serves as the source of sulfur atoms that are inserted into the octanoyl substrate [14]. Based on X- ray crystallographic studies carried out on a *Thermosynechococcus elongatus* homolog, a serine residue in the binding pocket for the auxiliary cluster is believed to serve as a fourth non-cysteinyl ligand to the auxiliary cluster, and was later supported in studies of the *E. coli* protein [12,15].

Mutations in LIAS and its trafficking protein result in low levels of lipoic acid that results in severe disease states due to the importance of lipoic acid in cellular metabolism. A required enzyme for the formation of lipoic acid, human LIAS has been linked to multiple mitochondrial dysfunction syndrome (MMDS). The connection between LIAS and MMDS was drawn from the observation that patients with the disease had low levels of lipoic acid, believed to result from the inactivity of LIAS [16]. However, MMDS is not caused by a mutation in LIAS, but instead is due to mutations in proteins involved in Fe–S cluster trafficking, such as NFU, ISCA2 and BOLA3 [16,17,18]. Another disease linked to LIAS, non-ketotic hyperglycinemia (NKH), is related to mutations in BOLA3, LIAS, and GLRX5, and is characterized by high levels of glycine in the blood, but has no impact on the respiratory chain and results in variable effects on iron levels within the cell [19]. The disease-causing mutant in the case of LIAS has been identified as an R249H point mutation, resulting in severe symptoms that include epilepsy in neonates, muscular hypotonia, and death of the patient at age four [20].

Much of the prior in vitro work with LIAS has been conducted with prokaryotic homologs. We have focused our studies on human LIAS and addressed the pathway for Fe–S cluster reconstitution of both the auxiliary and reducing cluster centers. These studies also provide an understanding of the downstream implications and molecular mechanisms that result in impaired function of human LIAS and subsequent disease states and complications. We have cloned, expressed, and purified a recombinant form of human LIAS (lacking the mitochondrial targeting sequence, residues 28–372) in *E. coli*. The isolated human LIAS was found to be functionally inactive in an enzymatic assay [14]. LIAS requires two [4Fe–4S] clusters for catalytic activity; therefore, we considered the possibility that the absence of catalysis reflected incomplete cluster reconstitution, a conclusion that was supported by iron quantitation experiments. To that end, we explored various methods of cluster reconstitution, with a particular focus on physiologically-relevant sources of mitochondrial Fe–S donor proteins, including ISCA1, ISCA2, GLRX5, BOLA3, ISCU, and the heterodimers BOLA3-GLRX5 and ISCA2-NFU. These were chosen because they had been implicated in the trafficking of clusters to LIAS based on experimental data or observations found in patients with NKH and MMDS [19], or to the reconstitution of other [4Fe–4S]-containing proteins [21,22]. Both full-length and truncated versions of ISCA2 were utilized as [2Fe–2S] donors to further compare the reactivity between the two constructs characterized in previous reports [23,24]. A BOLA3-GLRX5 heterodimer has also been shown to have [2Fe–2S] cluster trafficking ability and has been indirectly implicated in cluster transfer to LIAS [19,25,26]. Based on BOLA3’s capability of assisting in [4Fe–4S] cluster donations in yeast [27], *Saccharomyces cerevisiae* (*S. cerevisiae*) GLRX3 was also used as a control to confirm the reliability of the activity assay because it is a cytosolic protein and therefore would not be expected to reconstitute mitochondrial LIAS [28].

Accordingly, a number of potential [2Fe–2S] cluster donors were evaluated to determine if they were capable of delivering and assembling the requisite Fe–S cluster cores to LIAS to provide a fully active and functional enzyme. Variants of LIAS were also created using site-directed mutagenesis, which contained amino acid substitutions in one or other of each cluster binding site, to allow evaluation of a possible preferential ordering of cluster delivery. These mechanistic details were explored by use of kinetic and spectroscopic methods, including UV–Vis and electron paramagnetic resonance (EPR) spectroscopies, and revealed both a preference for cluster donor and a mechanistic ordering for reconstitution of the reducing and auxiliary cluster sites.

## 2. Results

### 2.1. Characterization of Native Recombinant and Substituted Derivatives of LIAS

**Figure 1:**
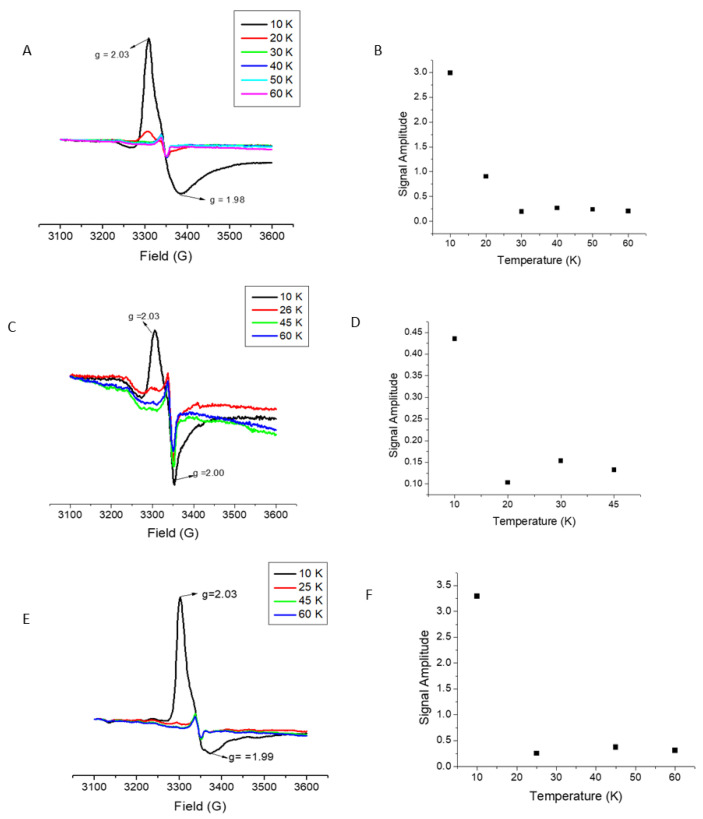
Shown above are the electron paramagnetic resonance (EPR) spectra obtained for reduced wild type lipoic acid synthesis (LIAS): (**A**) a single reducing cluster derivative; C137A (**C**); and the double reducing cluster derivative LIAS, C137A-C141A (**E**) are shown. The temperature dependence for each of the LIAS samples is shown beside their respective EPR spectra above in (**B**,**D**,**F**).

LIAS protein that had been purified but not undergone any Fe–S cluster reconstitution protocol, nor had any Fe–S cluster removed, was characterized by a variety of methods to determine the nature of the bound Fe–S cluster species in the as-isolated protein. Based on the results of EPR (Figure 1), UV–Vis, and iron quantitation data, it appears that immediately following purification, native recombinant LIAS is purified with one bound [4Fe–4S] cluster, in comparison to bacterial LIAS which purifies with seven equivalents of Fe and six equivalents of S, occupying or partially occupying both sites (Supplementary Figure S1, Supplementary Table S1) [9]. Characterization as a [4Fe–4S] center was confirmed by the temperature dependence of the EPR signal intensity for reduced clusters, which was observed to decrease significantly above 25 K; the observed g-values are also indicative of a [4Fe–4S] cluster.

**Figure 2:**
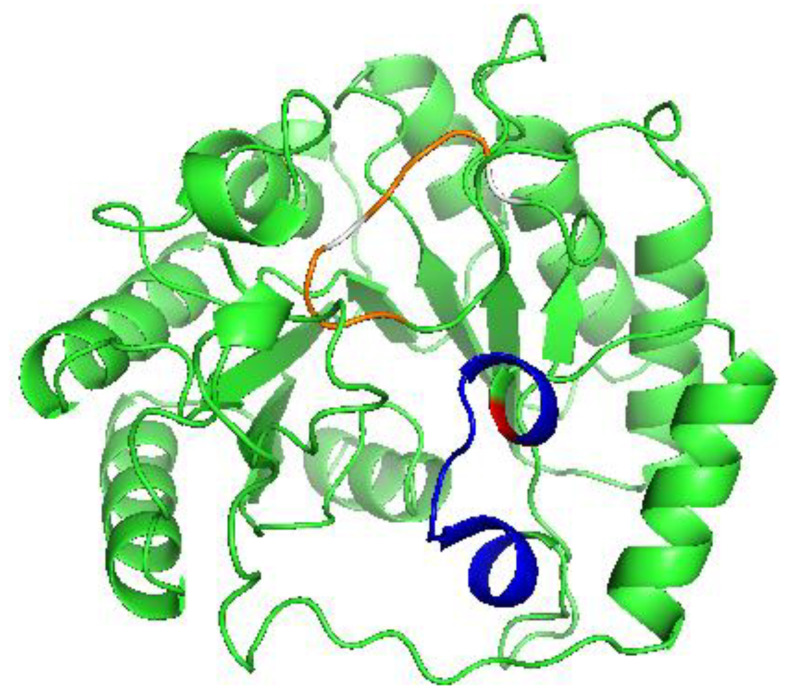
A model of human LIAS modeled using Phyre^2^ software. Highlighted in blue is the position of the auxiliary binding motif, with C106 highlighted in red. The location of the reducing cluster motif is shown in orange, with C137 and C141 highlighted in white.

Cys to Ala amino acid substitutions were made at each of the [4Fe–4S] cluster binding motifs to selectively eliminate binding at each of the reducing and auxiliary sites. The positions of these substitutions are highlighted in Figure 2. Two Cys to Ala mutations were made in the reducing cluster site, C137A and C137A-C141A, where the double Cys to Ala mutation, C137A-C141A, was made to confirm the loss of cluster binding ability at the reducing cluster site after it was found that C137A LIAS alone was able to bind a [4Fe–4S] cluster. While the EPR spectrum might also be indicative of an oxidized [3Fe–4S] cluster, both the temperature dependence of the EPR signal and the absorption spectrum are only consistent with a [4Fe–4S] center. By use of the same methods of characterization as the wild type LIAS, it was determined that both of the reducing cluster site derivatives were bound to a [4Fe–4S] cluster, similar to wild type LIAS (Figure 1). A Cys to Ala mutation was also made in the auxiliary cluster binding site, C106A. After purification, the C106A derivative was found to contain no Fe–S cluster based on EPR data and Fe quantitation, and that the auxiliary site derivative, C106A, lacked any bound cluster (Supplementary Figure S1, Supplementary Table S1, and Supplementary Figure S2). These results were also supported by iron quantitation, which yielded results consistent with native LIAS with retention of cluster for the reducing cluster derivative but loss of cluster for the auxiliary cluster derivative, and were also consistent with the as-isolated native LIAS and reducing cluster site derivative, having a cluster at the auxiliary site, but with complete cluster deficiency for the auxiliary cluster derivative.

The data obtained suggest an ordered model for cluster delivery with reconstitution of the auxiliary site as a necessary prerequisite for assembly/delivery of the cluster to the reducing site. Such a model is supported by EPR data (Figure 1) and iron quantitation, where the C137A and C137A-C141A derivative proteins (reducing cluster knockouts) bind a 4Fe–4S cluster, while the auxiliary cluster knockout (C106A) shows no evidence for a reducible bound cluster.

### 2.2. Evaluation of Cluster Donors for LIAS Reconstitution

Attempts to reconstitute LIAS further using previously described chemical reconstitution methods with FeCl_3_, and either L-Cysteine or Na_2_S as a source of sulfur, resulted in protein precipitation. However, fully reconstituted and active LIAS was obtained by use of certain cluster donor proteins, as detailed below. 

To assess formation of fully reconstituted LIAS, enzyme activity was determined by use of an established LC–MS assay [29] that monitored the formation of lipoic acid following double sulfur insertion to the assay substrate (*m/z* = 1010). The substrate used in the activity assay was a truncated version of the lipoyl carrier protein, the H-protein, where the octanoyl side chain was attached to the lysine through an amide bond. Native LIAS, as isolated, was unable to complete turnover and did not produce lipoic acid that could be detected via the LC–MS activity assay. This result is consistent with expectations for a partially reconstituted enzyme with only one bound [4Fe–4S] cluster, as noted earlier most likely positioned in the auxiliary site. Screens with multiple [2Fe–2S] protein donors and evaluation of LIAS activity by use of the LC–MS activity assay demonstrated that ISCU, and both the full-length and truncated versions of ISCA2 were able to reconstitute LIAS (Supplementary Figure S3). Double sulfur insertion into the octanoyl-containing substrate resulted in a product peak that was identified and quantified by LC–MS. Control experiments verified that product formation could only be facilitated by active, reconstituted LIAS. These [2Fe–2S] proteins have been linked to either upstream Fe–S trafficking or direct delivery and formation of the [4Fe–4S] clusters on LIAS [21,30,31].

ISCU cluster delivery resulted in the most active LIAS in the allotted formation time of 120 min, producing the largest amount of lipoyl product, followed by full-length ISCA2 (95 ± 10%, relative to ISCU promoted activity within the same time frame). Finding that ISCU functions as a donor to LIAS agrees with a previous study reported for *E. coli* LIAS [8], although the relevance of our result for human metabolism will be discussed later. The truncated construct of ISCA2 also showed modest capability to activate LIAS (55 ± 10%) but BOLA3 homodimer displayed negligible capacity to reconstitute LIAS. Other potential physiological [2Fe–2S] donors (Table 1) were unable to form functional LIAS in the LC–MS assay.

### 2.3. [Fe–S] Cluster Transfer Monitored via UV–Vis

In addition to activity assays, UV–visible spectroscopy was also used as a secondary screening method for cluster donors to provide a more complete view of the trafficking of Fe–S clusters to LIAS and its cluster binding mutations. This also provided confirmation that donors that were observed to produce an active form of LIAS were able to do so through Fe–S cluster transfer. Additionally, if a donor was able to produce an active form of LIAS and was observed to transfer an Fe–S cluster to LIAS via UV–Vis, then this also provided a direct measurement of the rate of cluster transfer from each of the cluster donor proteins to LIAS by monitoring absorbance change at 420 nm, a wavelength at which LIAS has a higher extinction coefficient (ε_420_ = 12,800 ± 2816 M^−1^cm^−1^) than the [2Fe–2S]-bound donor proteins [9]. Quantifying the rate constants for cluster transfer provides a better metric to elucidate those donors that are more relevant physiological candidates for Fe–S cluster donation to LIAS.

UV–Vis experiments were performed anaerobically, and addition of a holo-donor was used to initiate the transfer reaction. The same reactants used in the LC–MS assay were used for the activity assay, excluding the LIAS substrate. UV–Vis experiments were carried out anaerobically to minimize any cluster degradation that may interfere with accurate measurement of the kinetics of cluster transfer reactions.

(1) $$A=y_{0\;\;}+\;A_{0}\left(1-e^{-kt}\right)$$

**Figure 3:**
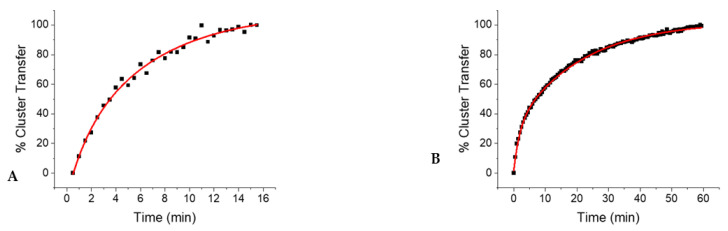
The relative extent of cluster transfer following the addition of holo-ISCA2 (**A**) and ISCU (**B**), was monitored over the course of 16 min to 1 h, as detailed in the experimental methods.

A single exponential growth equation, in the form of Equation (1), was used to fit the cluster transfer absorbance data where the absorbance of cluster changed over time, where *A* represents the absorbance at time *t*, *A*_0_ is the absorbance at time zero, and *k* is the observed rate constant. Absorbance data were converted to show the percent of cluster transferred by the donor over time (Figure 3) by normalizing the absorbance values from 0 to 100 percent, taking into account baseline correction.

Inasmuch as the auxiliary cluster site appeared to be almost completely occupied in the as-isolated form of the enzyme, cluster delivery must have taken place at the reducing site to yield the fully reconstituted and active form of human LIAS. Because formation of the [4Fe–4S] cluster at the reducing site is mediated by the consecutive delivery of clusters from [2Fe–2S] donors, the second step, forming the final [4Fe–4S] center, must be rate limiting and is consistent with prior observations for the [4Fe–4S] cluster protein, aconitase [24]. The highest apparent second order rate constant for cluster reconstitution was demonstrated by full-length ISCA2, followed by the truncated, but not necessarily physiological form of ISCA2, and then ISCU (Table 1).

### 2.4. Cluster Delivery to LIAS with Substitutions of Reducing and Auxiliary Cluster Ligands

To further test the model of consecutive reconstitution of the auxiliary cluster followed by the reducing cluster in human LIAS, cluster delivery to each of the reducing and auxiliary cluster knockout derivatives was examined. No cluster delivery was observed for the auxiliary cluster derivative, consistent with the requirement for auxiliary clusters to be present prior to assembly/delivery of the [4Fe–4S] reducing cluster. For the reducing cluster knockout derivative(s), limited and very modest rates of ~100–400 M^−1^min^−1^ of cluster transfer were found for ISCA2 and ISCU, most likely reflecting the predominantly populated auxiliary cluster site (as found for isolated native LIAS), with cluster addition to a minor population of enzyme that lacked the auxiliary cluster as isolated. 

## 3. Discussion

The results of our study provide additional insight into the cluster assembly pathway for human LIAS and other [4Fe–4S] cluster proteins, and provide a contrast for findings with bacterial homologs. Surprisingly, human NFU was unable to serve as an Fe–S cluster donor to LIAS. NFU has been implicated in MMDS1 because natural mutations promote the condition, while bacterial NFU has been reported to serve as a cluster donor to LIAS in *E. coli* [8,18,29]. Most likely, the mutations in NFU that give rise to MMDS prevent interaction with upstream partner proteins that are involved in trafficking an Fe–S cluster to the downstream LIAS target. A protein such as ISCA2 could then serve as the donor to LIAS, representing the case of MMDS4 [16]. ISCA2 could then be responsible for the observed low levels of lipoic acid if cluster transfer to LIAS needs to be mediated by human ISCA2 and NFU is unable to transfer a cluster to human ISCA2 [17]. It is also noteworthy that the post-translational modification database (dBPTM) indicates that LIAS has three possible post-translational modifications that would be absent in the *E. coli* counterpart. The three modifications are: a phosphotyrosine at the 20th amino acid; a phosphoserine at the 23rd amino acid; and an N6-acetyl lysine at the 318th amino acid. Of these three, only the N6-acetyl lysine would fall outside the mitochondrial targeting sequence, and it was found using structural modeling that the nucleotide portion of SAM would fall a distant 8 Å from this residue. 

ISCA1, which has been heavily implicated in [4Fe–4S] cluster formation and as a donor to [4Fe–4S] cluster proteins such as aconitase in *Saccharomyces cerevisiae*, was not found to be a viable [2Fe–2S] cluster donor to LIAS. However, it has been proposed that ISCA2 has a unique function within the mitochondria that bacterial homologs cannot replace, and is consistent with our observations [21]. It is possible, therefore, that one of the natural functions of ISCA2 is to mediate the reconstitution of LIAS. With respect to the connection to mutations in GLRX5 and BOLA3 [19] that cause non-ketotic hyperglycinemia, it is reasonable to conclude that GLRX5 and BOLA3 are most likely involved in up-field roles in cluster trafficking (either in homo or heterodimeric forms), but not in direct reconstitution of holo-LIAS.

Our LC–MS activity assays support previous findings that both [4Fe–4S] clusters are necessary for product turnover [13]. By use of EPR spectroscopy and iron quantitation, it appeared that binding of the auxiliary [4Fe–4S] cluster is independent of the reducing [4Fe–4S] cluster. However, the reducing cluster does not bind in the absence of the auxiliary cluster. This observation is supported by the lack of a [4Fe–4S] cluster EPR signal and iron quantitation for the auxiliary [4Fe–4S] cluster derivative, but both EPR and iron quantitation support the presence of the reducing [4Fe–4S] cluster in the reducing cluster derivative. If the auxiliary site is not occupied, the reducing cluster is unable to receive or bind a [4Fe–4S] cluster delivered via consecutive [2Fe–2S] delivery. The two motifs are also shown to be spatially close to one another, where the binding of the auxiliary cluster could potentially cause a structural change, enabling the reducing cluster to bind. This model is supported by the proposed movement of a serine residue that has been shown to be important in auxiliary cluster binding and implicates dynamic structural transitions that the protein must go through following binding of the auxiliary cluster [12,15].

The finding that ISCU and ISCA2, and to a lesser extent truncated ISCA2, can act as potential in vivo donors to LIAS provides further insight on the mechanism of reconstitution for [4Fe–4S] cluster proteins in a human system. It also provides additional evidence for the role of [2Fe–2S] trafficking in the reconstitution of [4Fe–4S] cluster proteins, and the apparent differences in these mechanisms between species. Additionally, our findings more clearly define the Fe–S proteins implicated in MMDS and NKH conditions that result, at least in part, from low levels of lipoic acid production arising from inactive LIAS.

## 4. Materials and Methods

### 4.1. Purchased Materials

Site directed mutagenesis was carried out using primers purchased from Integrated DNA Technologies, and Phusion DNA Polymerase and dNTPs were purchased from Fisher Scientific. Dithiothreitol (DTT), sodium dithionite L-cysteine, sodium sulfide, and ferric chloride were all purchased from Fisher Scientific, while PD-10 desalting columns were purchased from GE Healthcare. The peptide that served as an LIAS substrate (ESVKAASE, where an octanoyl group was attached lysine through an amide bond) was purchased from Biomatik.

### 4.2. Expression of LIAS

The sequence of human LIAS lacked the mitochondrial targeting sequence (residues 28–372). LIAS was PCR-amplified from a pGEM-3Zf(+) plasmid (gene provided by Dr. Johannes Mayr), and inserted into a pET28b(+) plasmid. Digestion of the plasmid took place at the NdeI and XhoI restriction sites, and the digested products were then extracted from an agarose gel using a Qiagen gel extraction kit and ligated using T4 DNA ligase. The ligation product was transformed into competent *E. coli* DH5α and BL21 (DE3) cells. Human LIAS was grown in 10 mL of Luria-Bertani (LB) broth media containing kanamycin (50 μM) at 37 °C overnight and then transferred to 3 L of fresh LB media containing kanamycin and incubated at 37 °C until the OD_600_ reached 0.8. Protein expression was induced by the addition of isopropyl β-D-1-thiogalactopyranoside (IPTG) (300 μM), and the cell culture was incubated at 30 °C for 10 h. Cell pellets were collected by centrifugation at 5000 rpm and held at −80 °C until purified. The substituted derivative versions of LIAS were also transformed into BL21 (DE3) cells and expressed in the same manner as the wild type LIAS.

### 4.3. Site Directed Mutagenesis

Amino acid substitutions to the reducing cluster binding site, C137A, and a double variant C137A-C141A, as well as the auxiliary cluster binding site, C106A, were prepared by PCR amplification. The primers used for the mutant PCR were made using Agilent QuickChange Primer Design and Phusion polymerase, purchased from Thermofisher. The PCR protocol included an initial denaturation at 98 °C for 1 min followed by 30 cycles of denaturation at 98 °C for 30 s, annealing at 55 °C for 30 s, and elongation at 72 °C for 3 min. After completion, the PCR was digested with Dpn1 (Fischer Scientific) at 37 °C for 2 h. The digested PCR reaction was then transformed into BL21-DE3 *E. coli* cells by use of the heat shock method and plated on kanamycin plates. Positive mutations were confirmed by sequencing through Genewiz.

### 4.4. Purification of LIAS

Both native LIAS and substituted derivatives were purified by use of the same method, where cell pellets were collected by centrifugation at 4330 g for 15 min, resuspended in 30 mL of 50 mM HEPES, 400 mM NaCl, pH 7.5, and lysed by use of a dismembranator. The lysate was centrifuged at 28,928× *g* for 30 min, the supernatant was subsequently applied to a Ni-NTA column, and the protein was eluted with a buffer containing 50 mM HEPES, 400 mM NaCl, 0.25 M imidazole, pH 7.5, prior to analysis on a 12% SDS-PAGE gel that was then visualized by Coomassie Blue staining. Protein concentration was determined by use of the Bradford assay.

### 4.5. Expression and Purification of Donor Proteins 

The full-length human NFU1 gene in the pET28b(+) plasmid was transformed into BL21 DE3 cells and over-expressed in *E. coli*, with purification as previously described [32], as well as human ISCA proteins [32]. Purification of *Hs* ISCU and *Thermatoga maritima* (*Tm*) NifS was performed as previously reported [33,34]. *Saccharomyces cerevisiae* GLRX3 (Δ1–35) and human GLRX5 (Q86SX6, residues 32–157) (Δ1–31), both lacking mitochondrial targeting sequences and expressed from pET28b(+) vector constructs in *E. coli* BL21 (DE3), were each purified using reported methods [33,34,35,36,37].

### 4.6. Iron Quantitation

A previously established protocol was utilized, where 60 μL of concentrated HCl was added to 25 μM samples of purified protein, as well as standard solutions of 1 to 200 μM FeCl_3_, and the samples were boiled at 100 °C for 15 min [38,39]. Samples were then centrifuged at 14,000 rpm for 2 min, after which 100 μL of supernatant was diluted with 1.3 mL of 0.5 M Tris-HCl, pH 8.5, and 0.1 mL of 5% sodium ascorbate, followed by 0.4 mL of 0.1% bathophenanthroline disulfonate. The samples were then incubated for 1 h at room temperature in the dark. The absorbance of each protein and an iron chloride control sample was measured at 535 nm on a UV–Vis spectrophotometer, and the concentration of Fe in each protein sample was determined using the calibration curve constructed using the absorbance values from the control samples.

### 4.7. UV–Vis Characterization

Observation of cluster transfer from the [2Fe–2S] protein donors to LIAS was performed in an anaerobic cuvette using 2.5 mM sodium dithionite, 0.5 mM DTT, 0.4 mM SAM, 3 mM glutathione, 125 µM LIAS substrate, 25 µM LIAS, and 100 µM holo-donor protein. The holo-donor was the last component added to the assay to initiate transfer. The wavelength range from 500 to 300 nm was then measured every 30 s over a period of 1 h at a scan rate of 600 nm/min. Because [2Fe–2S] cluster proteins generally have a lower extinction coefficient than [4Fe–4S] proteins, an increase in absorbance was expected as the cluster was transferred from the [2Fe–2S] donor to LIAS. Data were converted to show the percent of cluster transferred by the donor over time by normalizing the absorbance values from 0 to 100 percent, taking into account baseline correction.

### 4.8. LC–MS Activity Assays

LIAS activity assays were performed aerobically, according to a method described by Lanz et al., with dithionite, DTT, SAM, 3 mM glutathione, LIAS substrate, 25 µM LIAS, and excess donor protein [14]. The substrate used in the assay was a peptide (ESVKAASE) that mimics a truncated H-protein, with an octanoyl side chain attached to the lysine residue through an amide linkage [14]. After combining all the components and initiating the activity assay by addition of the holo-protein, the reaction mixture was maintained at 4 °C for 2 h. The reactions were quenched using 80 µL of 50% isopropyl alcohol and 20 µL 2M H_2_SO_4_ and then centrifuged for 20 min at 13,000 rpm. The samples were then analyzed by LC–MS to confirm the presence of a double (*m/z* = 1010) or single sulfur insertion (*m/z* = 978). LC–MS was completed with a Zorbax Rapid Resolution HT SB-C18 column (2.1 × 50 mm, 1.8 µm particle size). Solvent A was H_2_O with 0.1% formic acid and 1% acetonitrile, and B was acetonitrile with 0.1% formic acid and 10% H_2_O. The supernatant from the reaction mixture was separated with a gradient that began at 92% solvent A and 8% B, followed by an increase in solvent B to 19% during the first min that was maintained for 5 min. Subsequently, solvent B was decreased back to 8% over 1 min, and the column was allowed to re-equilibrate at these conditions for 3 min.

### 4.9. Preparation of Electron Paramagnetic Resonance (EPR) Samples

For analysis by EPR spectroscopy, samples of 25 μM wild type LIAS, and substituted derivatives at the reducing and auxiliary cluster binding sites, were reduced using 2 mM sodium dithionite and then frozen in 4 mm wall sample tubes. In experiments where the transfer of Fe–S clusters to LIAS were being analyzed, samples were incubated at 4 °C for 30 min with varying amounts of holo-ISCA2, 5 mM DTT, and 25 μM of purified LIAS, and were also treated with 2 mM sodium dithionite before freezing. Data were collected for each sample at temperatures ranging from 10 to 60 K, with a microwave power of 20 dB, and amplitude of 10 dB on a Bruker EMXPlus EPR with ColdEdge CryoStat.

## 5. Conclusions

In this study, it was found that the Fe–S donor proteins ISCA2 and ISCU were each able to transfer Fe–S clusters to wild type LIAS such that it was active and able to produce lipoic acid. ISCA2 appeared to transfer cluster most effectively and is the likely physiological donor, with ISCU serving a redundant role as a back-up if required. We have previously discussed the relevance and importance of redundancy in cluster trafficking and donor roles as a means of circumventing severe disease-causing mutations that debilitate regular carrier/donor activities along primary trafficking pathways [16,32,35,40,41].

These results are also consistent with the proteins previously suggested to promote formation of [4Fe–4S] clusters in downstream targets, and correlate with proteins where natural mutations have been implicated in patients found to have low levels of lipoic acid, resulting in severe disease states such as hyperglycinemia [16,19]. Most likely, mutations of other trafficking proteins, such as NFU can also result in low levels of lipoic acid because they form part of an Fe–S trafficking chain that donates clusters to one of the proposed LIAS donor proteins. An order of cluster addition is also proposed for wild type LIAS based on the ability of the reducing cluster derivatives to bind one [4Fe–4S] cluster, similar to the wild type, although no Fe–S cluster was able to bind to the auxiliary cluster derivative. Based on these findings, we propose that it is necessary for the auxiliary cluster site of LIAS to be occupied prior to reconstitution of the [4Fe–4S] reducing site.

## Figures and Tables

**Table 1:** Apparent second-order rate constants determined for the transfer of [2Fe–2S] clusters to as-isolated LIAS by various cluster donor proteins (including monomers, and homo- and hetero-dimers).

[2Fe–2S] Cluster Donor	Apparent Second Order Rate Constants
*H. sapiens* ISCA1	no transfer
*H. sapiens* ISCA2	3400 ± 200 M^−1^min^−1^
*H. sapiens* ISCA2 (truncated)	1130 ± 70 M^−1^min^−1^
*H. sapiens* NFU	no transfer
*H. sapiens* BOLA3	negligible transfer
*H. sapiens* ISCU	780 ± 50 M^−1^min^−1^
*H. sapiens* GLRX5	no transfer
*H. sapiens* Glrx5/BOLA3 heterodimer	no transfer
*S. cerevisiae* GLRX3	no transfer
*H. sapiens* Nfu/ISCA2 heterodimer	no transfer
*H. sapiens* ISCA1/ISCA2 heterodimer	no transfer

## Data Availability

Data are presented in this study or Supplementary Materials.

## Supplementary Materials

The following are available online at https://www.mdpi.com/1422-0067/22/4/1598/s1. Figure S1: UV–Vis spectra of wild type LIAS and mutants; Figure S2: EPR data and temperature dependence of C106A; Figure S3: Mass spectra of LIAS with and without the successful donor proteins, ISCA2 and ISCU; Table S1: Table of the molar equivalents of iron bound to wild type LIAS and its mutants.
