# Supplementary material for: Characterization and Reconstitution of Human Lipoyl Synthase (LIAS) Supports ISCA2 and ISCU as Primary Cluster Donors and an Ordered Mechanism of Cluster Assembly
Source: Int J Mol Sci. 2021 Feb 5;22(4):1598. doi: 10.3390/ijms22041598 (PMC7915201; doi:10.3390/ijms22041598)
Supplement: Supplementary file 1 [file ijms-22-01598-s001.pdf]

Supplementary Materials

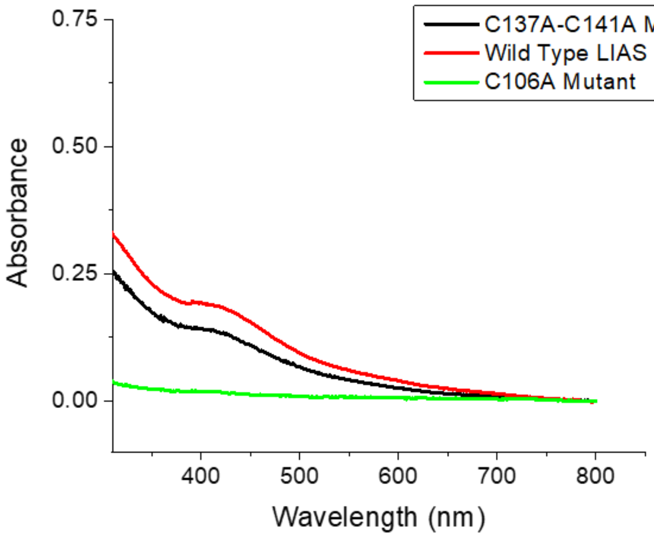

Figure S1. UV-Vis Spectra of wild type LIAS and mutants. Both wild type LIAS and the reducing cluster mutant C137A-C141A have the distinct peak at 420 nm indicating the presence of a [4Fe-4S] cluster. The auxiliary cluster mutant, C106A, lacks this feature indicating that an Fe-S cluster is not bound to the C106A mutation. Each of the samples contains 33 μM of protein.

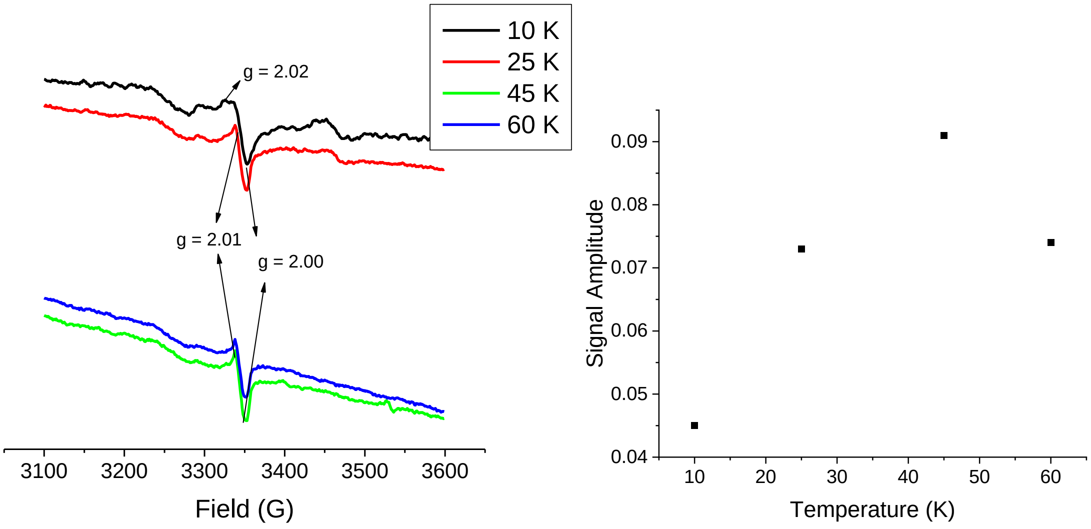

Figure S2. EPR data and temperature dependence of C106A. The EPR spectra shown above only show the free electron and lack an Fe-S cluster signal, this is supported by the temperature dependence shown on the right that does not follow the expected decrease in signal intensity that would be expected as the temperature increases.

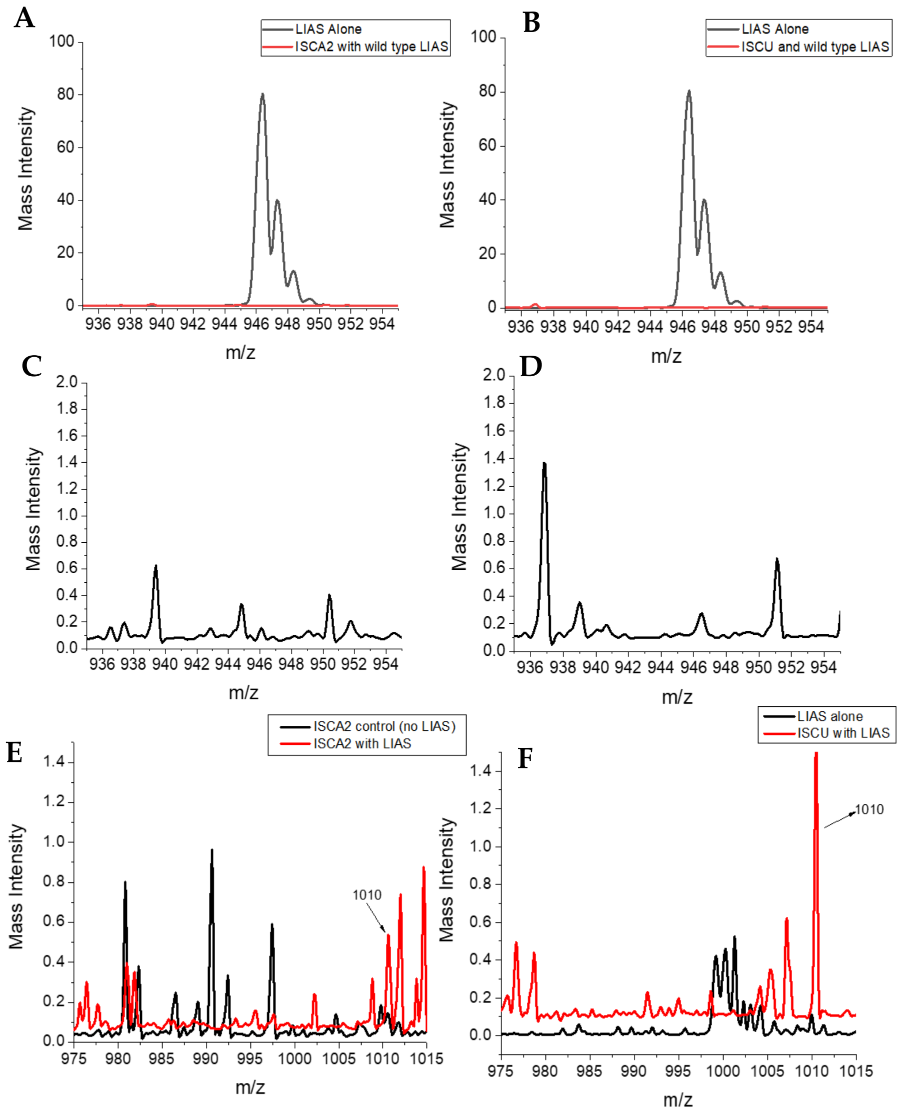

Figure S3. Mass Spectra of LIAS with and without the successful donor proteins, ISCA2 and ISCU. A. and **B** are mass spectra of wild type LIAS with (red) and without a donor protein (black) and with all the necessary reagents to carry out an LC-MS activity assay, **A** uses ISCA2 as a donor protein and **B** has ISCU as a donor protein. The peak shown in each spectrum at m/z = 946 is the unreacted octanoyl substrate peptide and can be seen prominently in the samples without a donor protein. **C** and **D** show the 946 mass spectrum region from **A** and **B** respectively, but without the prominent peak from the control of LIAS without a donor protein. **E** shows the double sulfur insertion product peak (m/z =1010) when ISCA2 is added (red) compared to when LIAS does not have ISCA2 added (black), **F** is the same information but with ISCU as a Fe-S cluster donor.

Supplementary Table 1. Table of the molar equivalents of iron bound to wild type LIAS and its mutants. Wild type and C137A both have at least 4 moles of protein bound to them indicating that a [4Fe-4S] cluster is bound to the majority of the protein in solution . C137A-C141A appears to still contain a [4Fe-4S] cluster based on the UV-Vis and EPR data, but less of the protein is bound to a [4Fe-4S] cluster species resulting in fewer moles of iron per mole of protein (**Figure S1** and **Figure 1**).

Protein	Moles of Fe per mole of protein
Wild Type LIAS	4.76
C137A LIAS	4.07
C137A-C141A LIAS	1.88
C106A LIAS	0.31
